# Child with progressive limb weakness caused by a neuroenteric cyst in the cervical segment: A case report

**DOI:** 10.1097/MD.0000000000046443

**Published:** 2026-05-12

**Authors:** Jiangli Wen, Qifan Hou, HaiBin Leng, Jing Yi

**Affiliations:** a Department of Neurosurgery, Changde Hospital, Xiangya School of Medicine, Central South University (The First People’s Hospital of Changde City), Changde, People’s Republic of China.

**Keywords:** case report, children, neuroenteric cyst, surgical resection

## Abstract

**Rationale:**

Neurenteric cyst is a rare congenital cystic lesion within the spinal canal that originates from the failure of the separation between the neural tube and the endoderm during the embryonic period. This disease is more commonly observed in the cervical and thoracic spinal cord. Pediatric cases are even rarer. The clinical manifestations are diverse. Early recognition and surgical intervention are crucial for the prognosis.

**Patient concerns:**

The male child was 3 years and 5 months old. He presented for medical attention due to “neck pain accompanied by limited mobility for 10 days” and was secondarily accompanied by weakness in the right upper limb and abnormal gait. The patient had a history of minor neck trauma history before the onset of the illness.

**Diagnoses:**

Magnetic resonance imaging shows a cystic lesion with low longitudinal relaxation time signal and high transverse relaxation time signal on the ventral side of the spinal canal at the cervical vertebra2–cervical vertebra 4 segments. There is no obvious enhancement, and neuroenteric cyst was suspected.

**Interventions:**

Subdural lesion resection and spinal canal plasty were performed under general anesthesia. During the operation, the cyst was located on the ventral side of the spinal cord, and the cyst wall was intact. The lesion was completely resected under the microscope.

**Outcomes:**

At the time of discharge, the muscle strength of the right upper limb had recovered to grade 3+ and that of the right lower limb to grade 4+. Neck pain was significantly relieved. Postoperative magnetic resonance imaging showed that the tumor was completely resected and the compression of the spinal cord was significantly relieved. The postoperative pathological diagnosis was consistent with a neuroenteric cyst.

**Lessons:**

This case suggests that although neuroenteric cysts are rare in children, their possibility should be considered in cases of neck pain accompanied by neurological dysfunction. Magnetic resonance imaging is the main diagnostic method, and complete surgical resection is currently the most effective treatment method. Intraoperative neuroelectrophysiological monitoring helps reduce the risk of neurological injury. The purpose and significance of this study lie in enhancing the understanding of neuroenteric cyst disease, improving the diagnostic rate, and reducing postoperative complications.

## 1. Introduction

Neurenteric cysts are a rare type of congenital cystic lesions of the central nervous system. It was first reported by Puusepp in 1934.^[1]^ Neurenteric cysts mainly originates from the failure of separation between the neural tube and endoderm during the early stage of embryonic development, resulting in residual endodermal epithelial cells in the spinal canal forming cysts.^[2]^ Such cysts are usually located on the ventral side of the spinal cord and are particularly prone to occur in the cervicothoracic segment. The clinical manifestations are diverse and include neck pain, limb weakness, and abnormal gait.^[1,3]^ Magnetic resonance imaging is the preferred method for diagnosing neuroenteric cysts. The typical manifestation is a cystic lesion in the spinal canal with a low Longitudinal Relaxation Time (T1) signal and high Transverse Relaxation Time (T2) signal without obvious enhancement.^[1,4]^ Given its extremely low incidence (only accounting for a very small proportion of spinal tumors),^[5,6]^ it is easily misdiagnosed as other cystic lesions, such as arachnoid cysts and epidermoid cysts.^[4]^ Pediatric cases are even rarer, and most of the literature reports are individual cases.^[1,7]^ However, there is still a lack of unified norms for diagnosis and treatment. This study reports a case of a neuroenteric cyst in a 3-year-and-5-month-old boy. Combining the clinical manifestations, imaging features, and surgical and pathological data, and referring to domestic and foreign literature, the diagnosis and treatment strategies are discussed.

## 2. Case report

**Figure 1.**
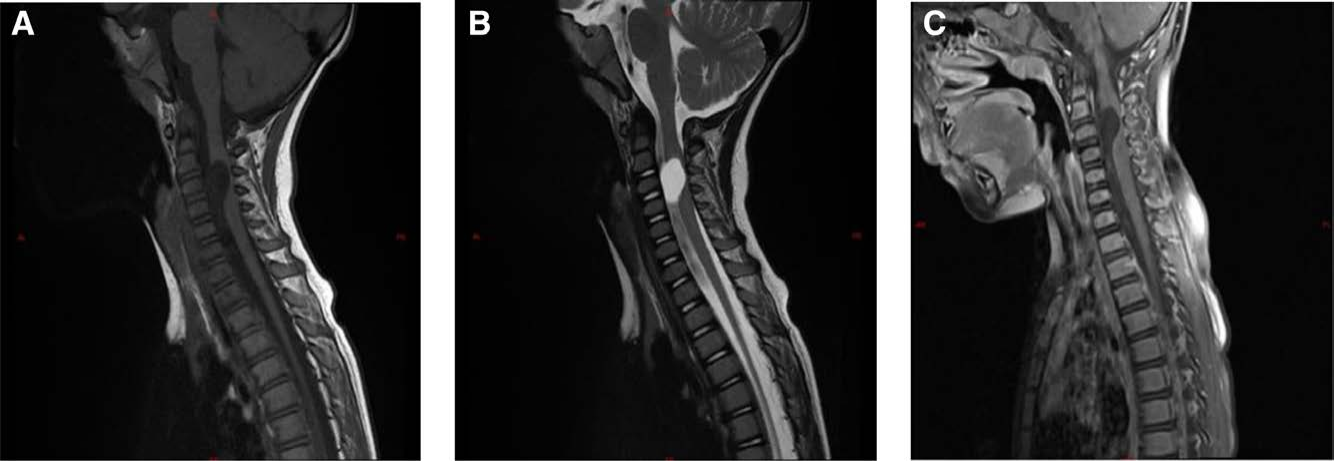
Preoperative magnetic resonance imaging imaging findings. (A) The sagittal T1-weighted image shows a hypointense lesion on the ventral side of the spinal canal at the cervical vertebra2–cervical vertebra 4 segments. The spinal cord is compressed and displaced posteriorly. (B) The sagittal T2-weighted imageshows that the lesion is hyperintense, and the dorsal side of the spinal cord is compressed and deformed. (C) The sagittal contrast-enhanced T1-weighted image shows that the lesion has no obvious enhancement, and the spinal cord is compressed. T1 = Longitudinal Relaxation Time T2 = Transverse Relaxation Time.

**Figure 2.**
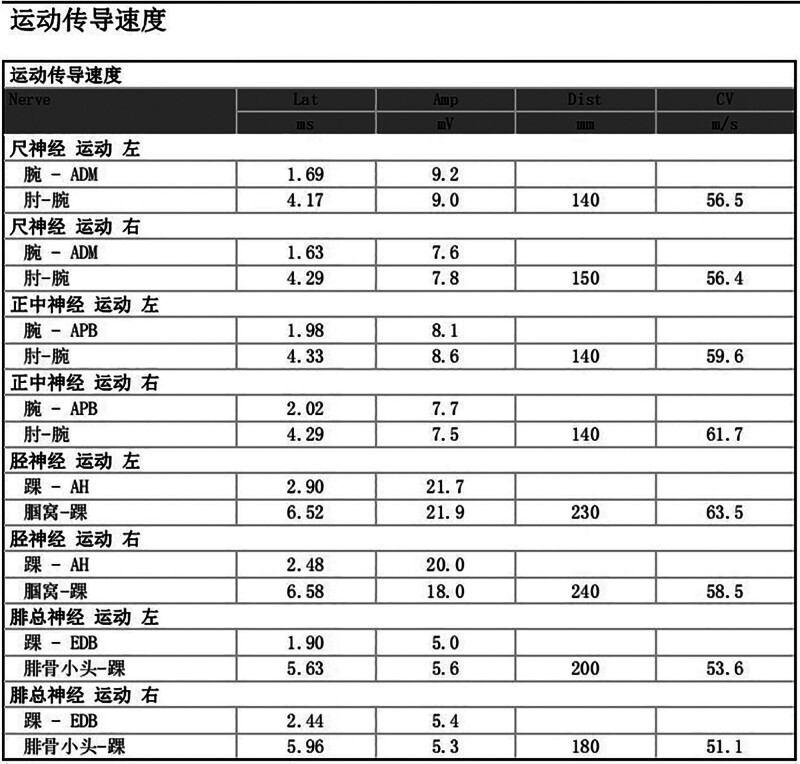
Results of preoperative electromyogram and evoked potential examinations. Motor nerve conduction examination revealed abnormalities in the amplitudes of nerve conduction and F-wave responses in the upper and lower limbs. The results of somatosensory evoked potential showed that the latency of bilateral Ninth Negative Peak was normal, but the latency of bilateral Thirteenth Negative Peak and Twentieth Negative Peak waves was prolonged, and the amplitudes were reduced, suggesting a conduction disorder at the level of the cervical spinal cord. A comprehensive judgment is consistent with the manifestations of high cervical spinal cord injury.

**Figure 3.**
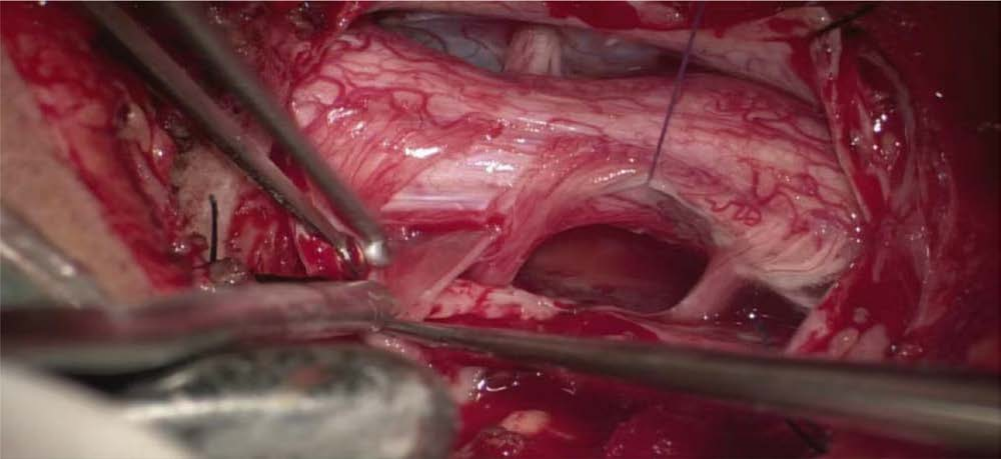
Microscopic image of intraoperative cyst resection. During the operation, the ventral spinal cord cyst was exposed using a microscope. The cyst wall was intact and the cyst fluid was slightly turbid. It could be seen that the cyst wall was pulled up and gradually separated from the spinal cord. Finally, complete resection was performed performed.

**Figure 4.**
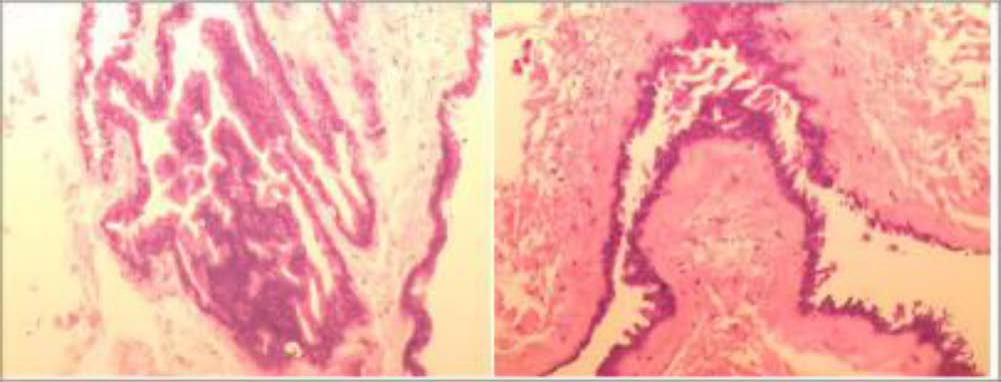
Postoperative pathological section findings: Hematoxylin and Eosin staining showed that the cyst wall was composed of pseudostratified ciliated columnar epithelium, and goblet cells could be seen in some parts, suggesting that it was derived from enterogenous epithelial tissue.

**Figure 5.**
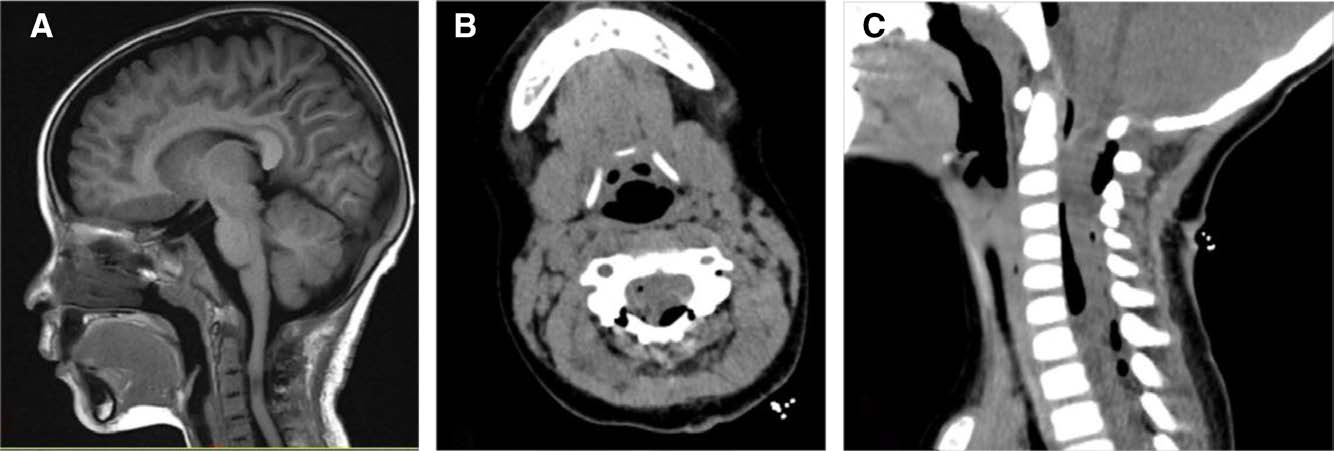
Postoperative imaging review results. (A) Postoperative sagittal T2-weighted magnetic resonance imaging showed complete resection of the lesion and significant relief of spinal cord compression. (B and C) Axial computed tomography and sagittal reconstruction images demonstrated good repositioning of the vertebral lamina, satisfactory reconstruction of the spinal canal structure, and no residual lesions were seen in the surgical area. T2 = Transverse Relaxation Time.

A 3-year-and-5-month-old boy was admitted to our department because of “neck pain accompanied by limited mobility for 10 days.” Ten days before admission, he developed neck pain and limited mobility without any obvious predisposing factors. Subsequently, weakness of the right upper limb gradually developed and progressed to weakness of the right lower limb. Upon further inquiry into the medical history, the child had a history of minor neck trauma before the onset of the illness. The child was one of the twins of the mother’s first pregnancy and second delivery, and his brother was in good health. Family members recalled that the child’s motor ability was slightly inferior to that of his brother when he was 2 to 3 years old. Physical examination upon admission revealed that the child was conscious. The muscle strength of the right upper limb was grade 1, and that of the right lower limb was grade 3. The left limb function was normal. Owing to pain, the child adopted a forced position (lying on the left side with the head tilted to the left). The neurological examination was not fully completed because of the child’s lack of cooperation. Magnetic resonance imaging revealed a cystic lesion with low T1 and high T2 signals on the ventral side of the spinal canal at the cervical vertebra 2–cervical vertebra 4 segments. It compresses the spinal cord, and no obvious enhancement is observed (Fig. 1). Electromyography and somatosensory evoked potential examinations indicated impaired function of the high cervical spinal cord (Fig. 2). Preoperative diagnosis: Space-occupying lesion in the cervical spinal canal (considered an enterogenous cyst), spinal cord embolism. After completing the preoperative preparations, the child underwent “Subdural lesion resection + Cerebrospinal fluid leak repair + Spinal canal plasty” under general anesthesia. During the surgery, under the assistance of a microscope, the cyst was dissected along the ventral side of the spinal cord. The cyst fluid was slightly turbid, consistent with the manifestations of an enterogenous cyst (Fig. 3). The cyst was completely resected. After the operation, the vertebral lamina was repositioned in situ, and the spinal canal structure was fixed and reconstructed. After the surgery, the child was transferred to the Neonatal Intensive Care Unit for observation. After extubation, the patient was transferred to the general ward. The postoperative pathological examination results were consistent with the histological characteristics of neuroenteric cysts (Fig. 4). Magnetic resonance imaging and computed tomography on the second day after the operation showed that the lesion was completely resected, and spinal cord decompression was satisfactory (Fig. 5). The child’s neurological function recovered well postoperatively. At the time of discharge, the muscle strength of the right upper limb was 3+ grade, and that of the right lower limb was 4+ grade at the time of discharge, and the pain was significantly relieved.

## 3. Discussion

The pathogenesis of neuroenteric cysts is generally considered to originate from the abnormal connection between the neural tube and primitive gut during the embryonic period, forming a residual neuroenteric canal, which later differentiates into a cystic structure similar to the intestinal epithelium.^[2]^ Pathologically, the cyst wall is mostly composed of pseudostratified ciliated columnar epithelium and goblet cells and has the ability to secrete mucus, which explains its compressive characteristics.^[1,8]^ These lesions grow slowly, and early symptoms are hidden. It is particularly difficult for young children to accurately describe their conditions. As cyst fluid accumulates due to secretion, the cyst gradually expands, causing chronic compression within the spinal canal. If there is minor trauma, acute neurological dysfunction may occur, exacerbating symptoms. Neurenteric cyst have certain imaging characteristics. Magnetic resonance imaging usually shows a cystic lesion on the ventral side of the spinal canal, with a low signal on T1-weighted images and high signal on T2-weighted images. The signal of the content is often equal to or slightly higher than that of cerebrospinal fluid.^[1,4]^ In some cases, mild compression of the spinal cord or spinal cord deformation can be observed at the site of the lesion. If necessary, enhanced scanning was used to rule out other solid lesions.^[3,4]^

In this case, the child’s magnetic resonance imaging revealed a cystic lesion on the ventral side of the spinal canal at the cervical vertebra 2–cervical vertebra 4 segments, compressing the spinal cord. This manifestation is consistent with the typical imaging features described above. Notably, there is a relationship between neuroenteric cysts and trauma. Although it is not a direct pathogenic factor, external force may cause the preexisting cyst to expand rapidly or displace, aggravating clinical symptoms.^[1,3]^

Surgical resection is the first-choice treatment for neurenteric cyst. Resecting the cyst wall as completely as possible can reduce the risk.^[9,10]^ However, if the cyst is severely adhered to the spinal cord, retaining part of the cyst wall to avoid nerve function injury is also an option.^[10]^ For ventral cysts, the choice of the surgical approach is of great significance. The anterior approach provided sufficient exposure and a high total resection rate. However, because it requires resection of the anterior column of the vertebral body, titanium cages and fusion fixation are often needed after the operation, which may have an adverse impact on the spinal development of children. Thus, the posterior approach is relatively safe. If the supraspinous ligament is retained during the operation and the lamina is reset through connecting plates, stability can be effectively maintained, making it suitable for young children. Although the exposure of the lateral approach has improved, the surgical field is still limited, and it often requires combined one-stage fusion treatment. Intraoperative electrophysiological monitoring, such as motor evoked potential and somatosensory evoked potential, is of great significance for positioning and functional protection.^[11]^ In the present case, microscopic dissection, spinal cord decompression, and dura mater repair were performed. Postoperative pathology confirmed it as an enterogenous cyst, and nerve function recovered well, indicating that early diagnosis and active surgical treatment are crucial for prognosis. Epidemiological studies have shown that the overall incidence of primary intraspinal tumors is relatively low, and it has been increasing annually with the popularization of magnetic resonance imaging and has gradually stabilized.^[5,12]^

## 4. Conclusion

As a rare subtype, neurenteric cyst requires enhanced clinical awareness to avoid misdiagnosis and missed diagnoses. In young children, if there is progressive limb weakness, especially when the upper limbs are the first to be affected, or when neurological symptoms deteriorate rapidly after minor trauma, neuroenteric cysts should be suspected. Regular follow-up should be performed after surgery. When symptoms recur, reoperation should be performed at an appropriate time, according to the age of the child and the characteristics of the lesion. The limitations of this study are that it only examines neurenteric cysts from the perspective of a single case, lacking universality. The diagnosis and treatment of neurenteric cysts are merely based on the experience of a single center, and there are deficiencies in the diagnosis and treatment process.

## Author contributions

**Investigation**: Jiangli Wen.

**Writing – original draft**: Qifan Hou, HaiBin Leng.

**Writing – review & editing**: Jing Yi.
